# Facile and Scale Up Synthesis of Red Phosphorus-Graphitic Carbon Nitride Heterostructures for Energy and Environment Applications

**DOI:** 10.1038/srep27713

**Published:** 2016-06-13

**Authors:** Sajid Ali Ansari, Mohammad Omaish Ansari, Moo Hwan Cho

**Affiliations:** 1 School of Chemical Engineering, Yeungnam University, Gyeongsan, Gyeongbuk 38541, South Korea; 2 Center of Nanotechnology, King Abdulaziz University, Jeddah 21589, Saudi Arabia

## Abstract

The development of heterostructured materials for efficient solar energy conversion and energy storage devices are essential for practical applications. In this study, a simple and relatively inexpensive method was used to improve the visible light-driven photocatalytic activity and electrochemical supercapacitor behavior of the graphitic carbon nitride (g-C_3_N_4_) by elemental red phosphorus (RPh). The as-prepared RPh-g-C_3_N_4_ was characterized in detail using a range of spectroscopic techniques to understand the structure, morphology, chemical interaction, and chemical state of the materials. The visible light-driven photocatalytic activity and supercapacitive electrode performance were assessed by the photodegradation of model colored, non-colored organic pollutants, and electrochemical half-cell measurements, respectively. The RPh-g-C_3_N_4_ heterostructure with 30 weight percent of RPh exhibited remarkably high photocatalytic activity for the degradation of pollutants compared to the bare constituent materials, which was further confirmed by the photoelectrochemical study under similar visible photoirradiation conditions. The RPh-g-C_3_N_4_ heterostructure supercapacitor electrode displayed a high capacitance of 465 F/g and excellent cyclic stability with capacitance retention of 90% after 1000 cycles at a current of 10 A/g. The superior performance was attributed mainly to the narrow band gap, high surface area, capacitive nature of RPh, and nitrogen-rich skeleton of g-C_3_N_4_.

The design and development of heterostructured photocatalysts, which are active and cover the maximum part of the solar energy spectrum, have attracted considerable research attention in current years^1^,^2^,^3^,^4^. Therefore, there have been many studies on semiconductor photocatalysts, in particular TiO_2_, for the visible light photocatalytic applications. However, TiO_2_ has a wide band gap (~3.2 eV), meaning that it cannot utilize the major part of the solar spectrum, and high photoinduced electron–hole (e^−^/h^+^) recombination rate^1^,^2^,^3^,^4^,^5^,^6^. Therefore, recent research has focused on developing highly visible light active photocatalysts, which is an alternative to TiO_2_^1^,^2^,^3^,^4^,^5^,^6^,^7^,^8^.

Electrochemical-based supercapacitors have attracted considerable attention for energy storage applications and are used widely in supercapacitor-based portable devices owing to their stability, rapid charging and slow discharging behavior, high power saving capability, and light weight^9^,^10^,^11^,^12^,^13^,^14^,^15^,^16^. The capacitive behaviors of the supercapacitors basically depend on the material textures and their important characteristics, such as stability, conductivity and theoretical capacitance. In recent years, carbon-based electrode materials for supercapacitor applications have attracted increasing attention because of their light weight, large surface area, conductive nature, and easy synthesis process^9^,^10^,^11^,^12^,^13^,^14^,^15^,^16^,^17^,^18^,^19^. However, their performance is found to be unsatisfactory for practical application and the capacitance decreases due to the insufficient penetration of electrolyte owing to the inert surface behavior. Therefore, to overcome this problem, nitrogen-rich, carbon-based materials have attracted attention because the presence of nitrogen improves the conductivity, capacity, and wettability of the supercapacitor electrode^20^.

For these purposes, tremendous efforts have been focused on polymeric-layered materials, particularly graphitic-like carbon nitride (g-C_3_N_4_), because it has a suitable band gap (~2.7 eV) for the effective utilization of visible light for the photocatalytic reaction^21^,^22^. The physicochemical stability owing to its cadenced CN framework and easy synthesis process make it suitable for a range of applications, including hydrogen production and the degradation of organic pollutants^21^,^22^,^23^,^24^,^25^. Despite this, the catalytic efficiency of g-C_3_N_4_ in a wide spectrum of the solar energy is still far from satisfactory because of the rapid photoinduced e^−^/h^+^ recombination rate and the limited specific surface area^24^,^25^,^26^,^27^. In addition to the above characteristics, g-C_3_N_4_ is also used in supercapacitor electrode material applications because of its high nitrogen content, easily tailorable structure, and low cost. The existence of nitrogen in the carbon based materials can improve the capacity while maintaining the excellent cyclic stability of the supercapacitor electrode. In addition, it also improves the wettability and expands the efficient exploitation of the surface^1^,^4^.

In recent years, elemental semiconductor photocatalysts, such as sulfur and red phosphorus (RPh), have been explored as highly visible light active photocatalysts because of their suitable band gap that can cover the maximum part of the solar spectrum^28^,^29^,^30^,^31^,^32^,^33^. Among them, RPh has attracted considerable attention because of its earth abundance, low cost, good stability, and suitable band position, and thus it can make an efficient heterojunction with other materials, such as nickel(II) hydroxide, carbon-based composite, yttrium(III) phosphate microspheres, etc., and enhance the life time of the charge carriers^29^,^34^,^35^. In addition, RPh is also a high capacitive and attractive anode material compared to conventional anodes because of its high theoretical capacity (~2596 mA h/g), which is almost seven times higher than graphite (~372 mA h/g)^36^,^37^,^38^,^39^,^40^. Owing to this high theoretical capacity of the RPh, it can be used as a charge accumulator. On the other hand, the high photogenerated e^−^/h^+^ recombination rate over RPh and the lower surface area has also restricted its high photocatalytic activity. Noble metal, black phosphorus, and carbon-based materials have been previously coupled with RPh, which acts as a supporting and/or co-catalyst to resolve this problem and decrease the recombination rate of charge carriers, leading to high photocatalytic activity^8^,^29^.

In view of the above discussion, it is worthy to construct a unique heterojunction of RPh with g-C_3_N_4_ by exploiting the unique physicochemical properties of both, e.g., suitable electronic band structure, lower band gap energy, high nitrogen content, and conducting behavior of g-C_3_N_4_, which helps to absorb large part of visible light from the solar spectrum and properties like narrow band gap and intensive visible light absorption ability as well as charge storage properties of RPh, which helps provide a large number of charge carriers on the surface that may participate in the photocatalytic reaction. Therefore, taking the advantage of these beneficial properties of both RPh and g-C_3_N_4_, and combing them in an optimum way, an highly effective and highly visible light driven photocatalyst and electrochemical supercapacitive electrode can be fabricated.

In this study, the RPh-g-C_3_N_4_ heterostructure was fabricated via a simple, safe, facile, and easily scaled up ball milling method for highly visible light driven photocatalysis and as a supercapacitor electrode material. The effects of the mechanical grinding time and weight ratio of the dopant were also studied. The as-prepared heterostructure showed significantly enhanced photocatalytic performance and photoelectrochemical behavior for the photodegradation of model colored and non-colored organic pollutants under visible light photoirradiation compared to the bare counterpart. The charge transfer mechanism over the photocatalyst was also proposed. Furthermore, the synthesized RPh-g-C_3_N_4_ heterostructure was assessed as an electrochemical supercapacitor electrode that exhibited an excellent specific capacitance of 465 F g^−1^ at a current density of 1 A g^−1^ with good retention up to 1000 cycles. The excellent performance shown by the RPh-g-C_3_N_4_ heterostructure compared to the bare counterpart was attributed to the suitable band gap position, high surface area, and conductive behavior, which highlights its potential applications in a range of fields.

## Results and Discussion

### XRD and UV-vis diffuse absorption analysis

**Figure 1:**
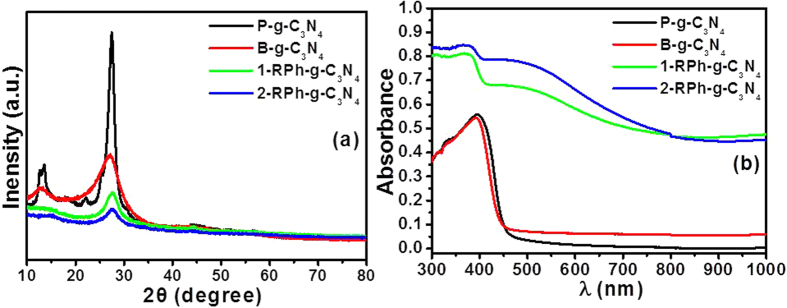
(**a**) XRD patterns and (**b**) UV-visible diffuse absorbance spectra of P-g-C_3_N_4_, B-g-C_3_N_4_, 1-RPh-g-C_3_N_4_, and 2-RPh-g-C_3_N_4_ heterostructure.

The phase composition and phase structure of as-prepared (P-g-C_3_N_4_), ball-milled (B-g-C_3_N_4_), 1-RPh-g-C_3_N_4_, and 2-RPh-g-C_3_N_4_ heterostructure were characterized by XRD, as presented in Fig. 1a. The appearance of a two feature XRD pattern of the as-synthesized g-C_3_N_4_ at ~13.06° and ~27.47° 2θ, were assigned to the interplanar stacking of the aromatic units and the crystal plane of the graphitic material, respectively^41^. After mechanical milling in the prepared g-C_3_N_4_ (P-g-C_3_N_4_), the width of the XRD pattern became broader, which may be due to the reduction of the size and number of layers. On the other hand, a significant change was also observed in the RPh-g-C_3_N_4_ heterostructure, where more broadening was observed after the mixing of RPh, which suggests a much greater reduction in size during mechanical milling of both constituents. This broadening confirms the reduction of the crystal size of the milled heterostructure according to the Scherer rule. On the other hand, the characteristic peak of the RPh was not observed in the case of the RPh-g-C_3_N_4_ heterostructure due to its low content and amorphous behavior. Zhu *et al*. reported that the mechanical grinding of g-C_3_N_4_ decreased the size significantly and resulted in the formation of thin g-C_3_N_4_ sheets. In the present case, a similar XRD pattern was also observed, which confirmed the decrease in size of the heterostructure^25^. Song *et al*. also reported in their RPh-graphene system that mechanical milling could easily reduce the size of the RPh material and peel off the layered material due to the high energy shear forces developed during the mechanical process^39^. Therefore, based on these explanations, it is believed that the size of the commercial RPh is broken down to a smaller size and stacked g-C_3_N_4_ sheets are formed simultaneously through the mechanical grinding of the RPh and as-prepared g-C_3_N_4_ in the ball milling jar^39^.

The influence of the RPh on the physiochemical properties of g-C_3_N_4_ was analyzed by the UV-vis diffuse absorbance/reflectance spectroscopy, which are depicted in Figs 1b and S1. The fundamental absorption edge of the as-prepared and milled g-C_3_N_4_ occurred at ~450 nm, which is the characteristic absorption wavelength of g-C_3_N_4_^41^. The RPh-g-C_3_N_4_ heterostructures showed significantly enhanced absorption in the visible region accompanied by a shift in the red absorption edge compared to the as-prepared P-g-C_3_N_4_ and milled B-g-C_3_N_4_, which might be due to the combined synergistic effects of the narrow band gap and light harvesting characteristics of RPh and g-C_3_N_4_^6^,^22^,^42^. These results indicate that the RPh-g-C_3_N_4_ heterostructure is active in the wide visible light range and may produce photoinduced charge carriers under visible light photoirradiation, which is highly favorable for the photocatalytic reactions.

### TEM analysis

**Figure 2:**
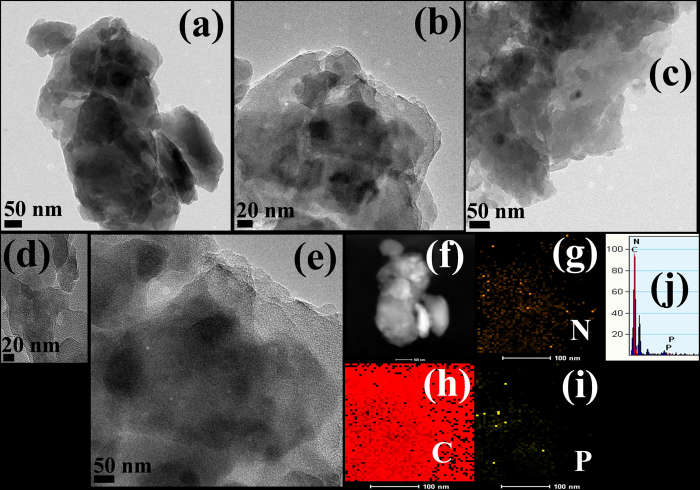
(**a–e**) TEM images of the representative 2-RPh-g-C_3_N_4_ heterostructure at different magnifications, (**f–i**) scanning transmission electron microscopy elemental mapping, and (**j**) EDX of the 2-RPh-g-C_3_N_4_ heterostructure.

The morphology of the as-synthesized representative samples was observed by TEM and the results are presented in Fig. 2. Figures S2 and S3 show the TEM image of B-g-C_3_N_4_, which shows a thick and agglomerated sheet-like structure. The TEM image of the 2-RPh-g-C_3_N_4_ heterostructure at different magnifications which is different from the B-g-C_3_N_4_, clearly shows the RPh particles with irregular shapes on the g-C_3_N_4_ sheet (Fig. 2a–e). Energy-filtered transmission electron microscopy and corresponding element mapping were conducted and the results in Fig. 2f–i further show that the carbon, nitrogen, and phosphorus can be found over the samples, which indicate the presence of phosphorus in the heterostructure. Figure 2j presents the EDX spectra of the 2-RPh-g-C_3_N_4_ heterostructure, which further ensures the presence of phosphorus in the representative sample. These results strongly confirm that the g-C_3_N_4_ and RPh are attached closely to form heterostructures that are expected to be helpful for various studies and applications. Similar TEM images were also observed in the previous RPh-carbon based nanocomposites^38^,^39^,^40^.

### XPS analysis

**Figure 3:**
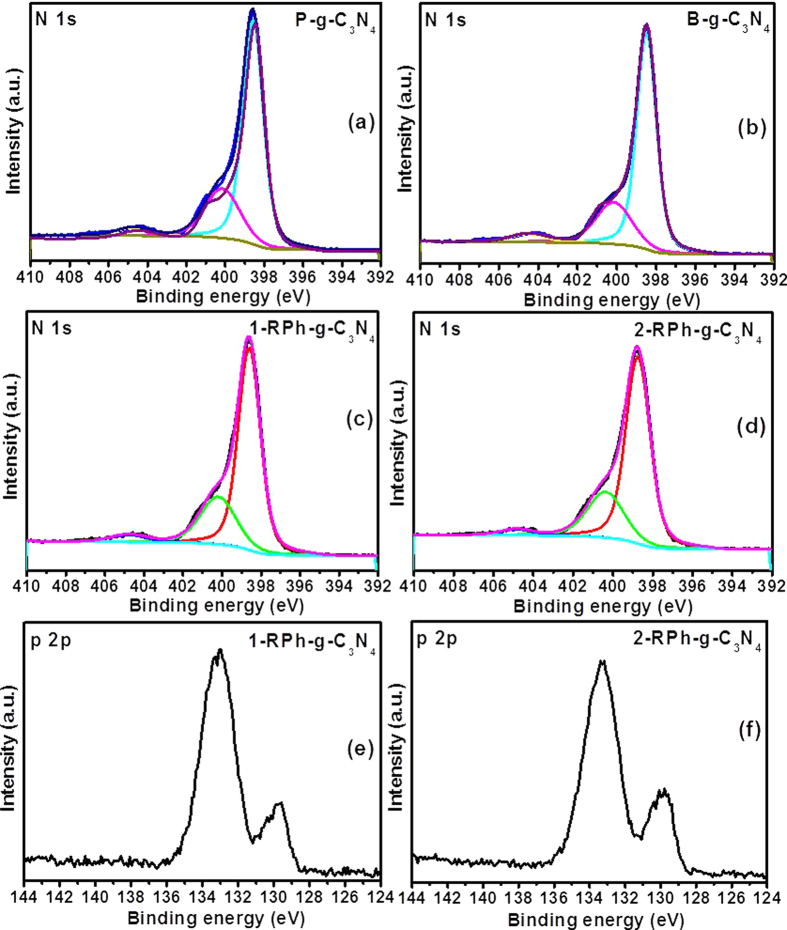
High resolution N 1s core level spectra of (**a**) P-g-C_3_N_4_, (**b**) B-g-C_3_N_4_, (**c**) 1-RPh-g-C_3_N_4_, (**d**) 2-RPh-g-C_3_N_4_ heterostructure, whereas (**e**) is the P 2p core level spectra of 1-RPh-g-C_3_N_4_ and (**f**) 2-RPh-g-C_3_N_4_ heterostructure.

XPS was performed to examine the surface composition and chemical bonding between the different constituents present in the prepared samples, as presented in Figs 3, S4 and S5. The full line spectra of the P-g-C_3_N_4_, B-g-C_3_N_4_, 1-RPh-g-C_3_N_4_, and 2-RPh-g-C_3_N_4_ confirmed the presence of C, N, and P (Fig. S4). Figure S5a–d shows the C 1s high resolution spectra of P-g-C_3_N_4_, B-g-C_3_N_4_, 1-RPh-g-C_3_N_4_, and 2-RPh-g-C_3_N_4_ heterostructures, which shows two distinct peaks at 284.53 and 287.99 eV due to the two different chemical state of carbon, i.e., sp2 hybridization and C-N bonding present in the samples^22^,^41^. Figure 3a–d shows the N 1s high resolution core level spectra of P-g-C_3_N_4_, B-g-C_3_N_4_, 1-RPh-g-C_3_N_4_, and 2-RPh-g-C_3_N_4_ with the corresponding deconvoluted Gaussian fitted peaks. The fitted deconvoluted spectra revealed the existence of the distinguishable chemical bonding mode of nitrogen species, such as the peaks located at ~398.49 eV, ~400.15 eV, and ~404.49 eV were assigned to the triazine ring, tertiary nitrogen, and the charge effect, respectively^22^,^43^. Furthermore, a comparison of the dominant peak at ~398.6 and ~398.75 for 1-RPh-g-C_3_N_4_ and 2-RPh-g-C_3_N_4_, respectively, showed that the binding energy (BE) was shifted to a higher energy compared to the P-g-C_3_N_4_ (~398.49 eV) and B-g-C_3_N_4_ (~398.5 eV). These higher shifts in the BE were attributed to the electronic interactions occurring between RPh and g-C_3_N_4_ during the high energy ball milling, which are helpful for enhancing the properties of the resulting heterostructure^21^.

Figure 3e,f presents the P 2p photoelectron peak of the 1-RPh-g-C_3_N_4_ and 2-RPh-g-C_3_N_4_ heterostructure, in which the two characteristic peak at 129.49 ± 0.01 and 130.03 ± 0.01 eV for 1-RPh-g-C_3_N_4_, and 129.58 ± 0.01 and 130.35 ± 0.01 eV for 2-RPh-g-C_3_N_4_, were assigned to the doublets of P 2p_3/2_ and P 2p_1/2_. The most dominant peak at ~133.4 ± 0.01 eV indicated the presence of pentavalent form of phosphorous in the representative samples. In addition, the photoelectron peak at 130.35 ± 0.01 eV, which was more dominant in the case of the 2-RPh-g-C_3_N_4_ heterostructure, was assigned to the existence of a P-C bond, which may be due to the substitution of some constituents in g-C_3_N_4_^22^. These results are also in accordance with the previous observed results^10^,^22^. Furthermore, in the case of P 2p photoelectron peak, the BE of P 2p in the case of the 2-RPh-g-C_3_N_4_ nanohybrids was red shifted compared to 1-RPh-g-C_3_N_4_. This provides a clear evidence of the electronic interactions between the RPh and g-C_3_N_4_, which modifies the structural and optical properties^22^.

### Visible light photocatalytic activity and its proposed mechanism

**Figure 4:**
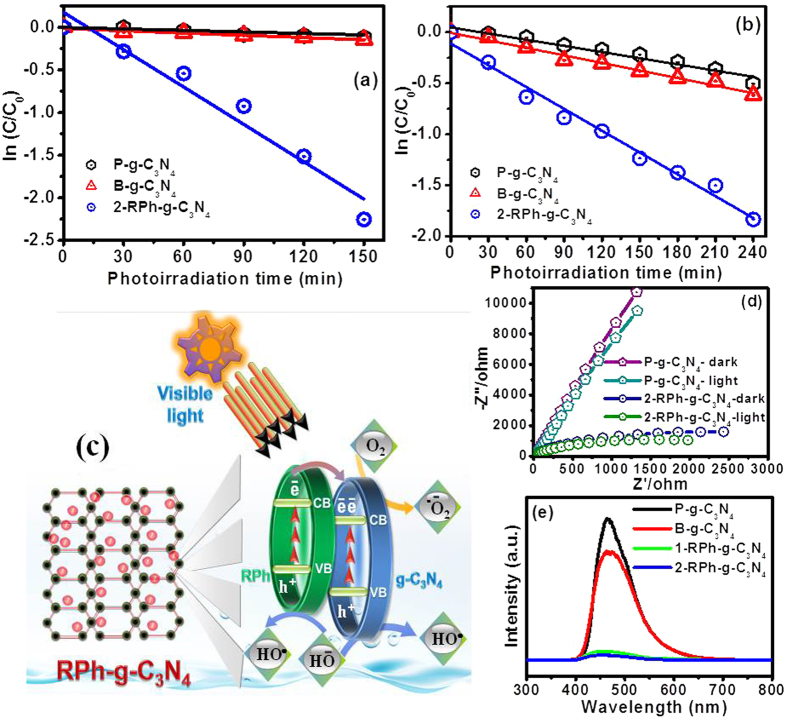
(**a**) Degradation kinetic plot of (**a**) MO and (**b**) RhB as a function of the visible light photoirradiation time by the P-g-C_3_N_4_, B-g-C_3_N_4_, and 2-RPh-g-C_3_N_4_ heterostructure photocatalyst, (**c**) proposed schematic diagram for the illustration of photoexcitation of the charge carriers, separation, and movement process in the presence of light on the 2-RPh-g-C_3_N_4_ heterostructure interface under visible photoirradiation, (**d**) EIS spectra of the representative photocatalysts in the dark and under visible photoirradiation, and (**e**) PL spectra of the P-g-C_3_N_4_, B-g-C_3_N_4_, 1-RPh-g-C_3_N_4_ and 2-RPh-g-C_3_N_4_ heterostructure.

To further examine the polymeric skeleton alteration of g-C_3_N_4_ after the addition of RPh and its associated interaction, a model organic pollutant, which is used frequently in the textile industry was chosen and a photocatalytic experiment was set up under visible light photoirradiation. The direct photolysis of the organic pollutant was also performed under illumination conditions and the result showed that the negligible photolysis had occurred^42^. Figures 4a,b and S6 presents the photodegradation ability of P-g-C_3_N_4_, B-g-C_3_N_4_, 1-RPh-g-C_3_N_4_, 2-RPh-g-C_3_N_4_, 3-RPh-g-C_3_N_4_, and 4-RPh-g-C_3_N_4_ heterostructured photocatalysts for the degradation of RhB and MO as a function of the irradiation time. The P-g-C_3_N_4_, and B-g-C_3_N_4_ photocatalysts showed low activity, which indicated that it is a poor photocatalyst under visible photoirradiation, whereas 1-RPh-g-C_3_N_4_ and 2-RPh-g-C_3_N_4_ heterostructure photocatalysts showed reasonable degradation efficiency under similar photoirradiation conditions. In particular, the 2-RPh-g-C_3_N_4_ heterostructures photocatalysts showed the highest photocatalytic ability for the degradation of MO and RhB under visible photoirradiation. For a better understanding of the photocatalytic activity of each photocatalyst, the apparent rate constant (k) was obtained using the methodology reported elsewhere^42^,^44^. Figures 4a,b, and S6 presents a ln(C_0_/C) kinetic plot of the organic pollutant as a function of the photoirradiation time. As shown in Figs 4a and S6a, under visible photoirradiation, the apparent *k* for the degradation of MO over the P-g-C_3_N_4_, B-g-C_3_N_4_, 1-RPh-g-C_3_N_4_, 2-RPh-g-C_3_N_4_, 3-RPh-g-C_3_N_4_, and 4-RPh-g-C_3_N_4_ heterostructures were calculated to be 0.00087, 0.00088, 0.00189, 0.01462, 0.00058, and 0.0026/min, respectively. The 2-RPh-g-C_3_N_4_ heterostructure photocatalysts showed much higher rate constant than the P-g-C_3_N_4_ and B-g-C_3_N_4_. Similarly, the apparent *k* for the degradation of RhB over the P-g-C_3_N_4_, B-g-C_3_N_4_, 1-RPh-g-C_3_N_4_, 2-RPh-g-C_3_N_4_, 3-RPh-g-C_3_N_4_, and 4-RPh-g-C_3_N_4_ heterostructures were 0.0020, 0.0024, 0.0030, 0.0071, 0.00023, and 0.0012/min, respectively. From the results it can be seen that the optimized photocatalyst showed higher degradation rate than its bare constituent materials. The above differences in rate constant for the degradation of MO and RhB may be due to the complex structure of RhB compared to the MO, which increases the steric hindrance and generates complexity during the reaction with the reactive photogenerated radicals^45^.

The colored organic pollutant can have a sensitization effect and its degradation via the sensitization pathway may be possible. Therefore, to ensure the precise photocatalytic activity with the exclusion of the sensitization effect, non-colored phenolic 4-NP compound was used to assess the intrinsic photocatalytic activity of the optimized heterostructured photocatalyst under visible photoirradiation (Fig. S7). The *k* values of the P-g-C_3_N_4_ and 2-RPh-g-C_3_N_4_ heterostructures for the degradation of 4-NP were calculated to be ~0.0014 and ~0.005/min, respectively, showing that the 2-RPh-g-C_3_N_4_ exhibits higher rate constant than P-g-C_3_N_4_. This provides solid support for the intrinsic photocatalytic activity of the prepared photocatalysts because the organic phenolic 4-NP is colorless and cannot sensitize under visible light irradiation.

In order to provide an insight to the enhanced photocatalytic performance of the 2-RPh-g-C_3_N_4_ nanohybrid photocatalysts, a tentative systematic illustration of the expected and the possible excitation of the charge carriers, their transfer mechanism for the photocatalytic activity of 2-RPh-g-C_3_N_4_ nanohybrid under visible light irradiation were proposed based on the band edge energy. The P-g-C_3_N_4_ and B-g-C_3_N_4_ exhibited poor photocatalytic activity under visible light photoirradiation. Thus it can be interpreted that although g-C_3_N_4_ has a narrow band gap, only a small fraction of charge carriers participated in the reaction and other photo generated electrons holes tended to recombine during the reaction. In comparison to these, the photocatalytic result of the 2-RPh-g-C_3_N_4_ heterostructure displayed superior photocatalytic activity for the degradation of the colored organic pollutants and non-colored phenolic compounds under similar photoirradiation conditions.

The enhanced photocatalytic activity of 2-RPh-g-C_3_N_4_ can be explained on the basis of the edge potential of the conduction band of the g-C_3_N_4_, which is more negative than RPh, and thus is helpful for the movement of the excited CB electron of g-C_3_N_4_ to the CB of the RPh through the heterojunction under visible photoirradiation^43^,^46^. Similarly, the photo-induced holes on VB of the RPh can also jump to the VB of the g-C_3_N_4_ (Fig. 4c). This simultaneous migration and transfer of photogenerated electrons and holes through the heterojunction is helpful in reducing the probability of the recombination of photogenerated charge carriers and also provides a large amount of electrons and holes over the surface of the photocatalyst, which helps to further enhance the photocatalytic activity of these materials^6^,^8^,^22^,^41^,^43^. The available charge carriers over the surface of the 2-RPh-g-C_3_N_4_ nanohybrid promoted the series of oxidative and reductive reactions responsible for the degradation of the pollutant under visible light irradiation. The photogenerated and excited electrons located on the surface of the nanohybrid were then trapped by the oxygen molecules dissolved in water to yield the superoxide radical anion (^•^O_2_^−^), whereas the holes located on the surface reacted with the surface adsorbed hydroxyl ions to form highly reactive HO^•^. These highly reactive radicals are responsible for the photodegradation and mineralization of pollutants^4^,^5^,^8^,^24^.

To further elucidate the effective separation of the photo-induced charge carrier and charge transfer resistance over the surface of the 2-RPh-g-C_3_N_4_ heterojunction, a further EIS approach was conducted in the dark and under visible photoirradiation, and the results are presented in Fig. 4d. A smaller semicircular arc in the Nyquist plot indicates lower electron transfer resistance, effective separation, and easy transportation of photogenerated electron-hole pairs^21^,^23^,^24^,^41^. The impedance spectra of the 2-RPh-g-C_3_N_4_ heterostructure under visible photoirradiation reflects the smaller semicircular arc compared to the P-g-C_3_N_4_, which indicates that the enhanced photo excited electron/hole separation and faster interfacial charge transfer occurred over the surface of the 2-RPh-g-C_3_N_4_ nanohybrid under visible light irradiation. In addition to EIS, PL was also used as a complementary technique to evaluate the fate of the electron-hole pairs, efficiency of charge carrier trapping, and recombination of electron holes pairs on the surface of the photocatalyst^4^,^5^,^6^,^24^,^41^. The intensity of the PL spectra generally reflects the recombination process of the photoinduced charge carriers. A high emission intensity indicates rapid charge recombination of the photogenerated charge carriers, whereas a weaker PL intensity indicates a lower rate of electron-hole recombination, which is favorable for enhancing the photocatalytic activity of the materials. The PL spectra of the 2-RPh-g-C_3_N_4_ heterostructures revealed similar weaker peaks compared to the other photocatalysts, which is depicted in Fig. 4e. This might be due to the lower recombination rate of the photogenerated electrons and holes, which are proposed to contribute to the enhanced photocatalytic activity over the surface of the photocatalyst. These results reveal an analogous trend with respect to the above discussion and photocatalytic results.

The capacitive behavior of the RPh in 2-RPh-g-C_3_N_4_ heterostructure may also play an important role in enhancing the photocatalytic activity of the nanohybrid. RPh has the highest theoretical storage capacity, which enables the charge storage properties that can greatly improve charge separation in the present heterostructures. Therefore, the excellent photocatalytic activity of 2-RPh-g-C_3_N_4_ may be due to the presence of excess charge carriers on the surface of the photocatalyst which drives a series of reactions to eventually give highly reactive radicals, responsible for the photodegradation of the organic pollutants. This capacitive behavior of the 2-RPh-g-C_3_N_4_ hybrids might be induced by RPh, which provide an excess of charge carriers in the presence of visible light over the surface of the photocatalysts through series of excitation process as mentioned above. Due to all these reasons the 2-RPh-g-C_3_N_4_ heterostructure showed excellent photocatalytic activity compared to the other materials under visible photoirradiation.

The photocatalytic degradation reaction occurs on the surface and thus the study of the surface area 2-RPh-g-C_3_N_4_ heterostructure may also give an idea of the possible mechanism taking place which accounts for its high photocatalytic activity. The surface area of the photocatalysts was estimated from the N_2_ adsorption-desorption isotherm and the measured Brunauer-Emmett-Teller specific surface area of the P-g-C_3_N_4_, B-g-C_3_N_4_, and 2-RPh-g-C_3_N_4_ heterostructures were 3.4846, 3.5468, and 11.4404 m^2^/g, respectively. Thus it can be seen that P-g-C_3_N_4_ and B-g-C_3_N_4_ exhibit a small surface area, whereas in comparison the 2-RPh-g-C_3_N_4_ heterostructure exhibited a ~3.2 fold higher surface area. The better photocatalytic performance of the 2-RPh-g-C_3_N_4_ heterostructure was attributed to the high surface area because the larger surface area of materials provides a good contact between the organic pollutant and the photocatalyst, hence is favorable for photocatalytic reactions. Thus it can be concluded that the excellent and remarkably enhanced photocatalytic activity of the well-designed 2-RPh-g-C_3_N_4_ heterostructure is due to the combine synergistic effect of the reduced band gap, difference in the band edge potential, high specific surface area, good conductive behavior and the effective separation of photogenerated electron holes through the heterojunction.

### Electrochemical supercapacitive behavior of the RPh-g-C_3_N_4_ heterostructure

**Figure 5:**
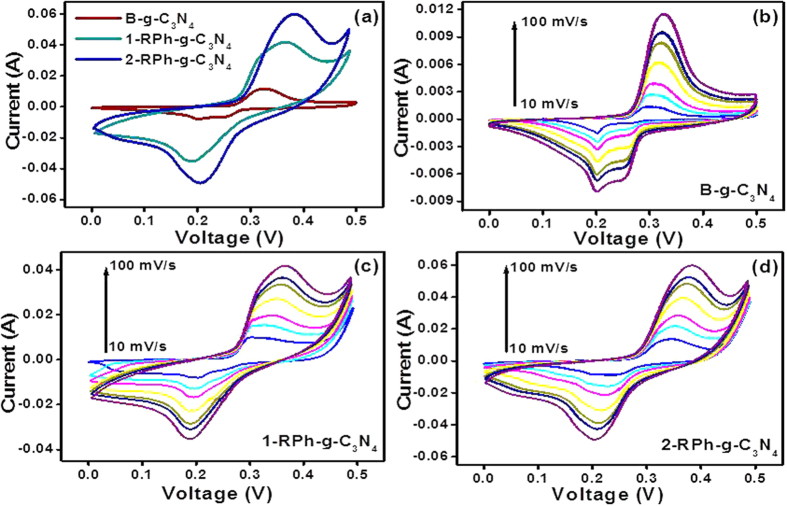
(**a**) Comparative cyclic voltammogram of B-g-C_3_N_4_, 1-RPh-g-C_3_N_4_, and 2-RPh-g-C_3_N_4_ at a scan rate of 100 mV s^−1^, (**b**) cyclic voltammogram of B-g-C_3_N_4_ at a scan rate of 10–100 mV s^−1^, (**c**) cyclic voltammogram of 1-RPh-g-C_3_N_4_ at a scan rate of 10–100 mV s^−1^, and (**d**) cyclic voltammogram of 2-RPh-g-C_3_N_4_ at a scan rate of 10–100 mV s^−1^.

To further understand the improvement in the electrochemical supercapacitive performance of the altered nitrogen-rich g-C_3_N_4_ by the introduction of RPh, cyclic voltammetry (CV) and the galvanostatic charge-discharge (GCD) technique were performed, which are used widely to determine the specific capacitance of materials. Figure 5a presents the comparative cyclic voltammograms of the B-g-C_3_N_4_, 1-RPh-g-C_3_N_4_ and 2-RPh-g-C_3_N_4_ heterostructures at a fixed scan rate of 100 mV s^−1^. It must be added that in all the heterostructures, the g-C_3_N_4_ is the main matrix and the BET surface area and photocatalytic performance of P-g-C_3_N_4_ and B-g-C_3_N_4_ were found to be similar. Therefore, in all these electrochemical supercapacitive experiments, the measurements of the optimized heterostructures were compared with only B-g-C_3_N_4_. The comparative CV showed that the 2-RPh-g-C_3_N_4_ heterostructure displayed a large capacitive area and better electrochemical supercapacitive performance than B-g-C_3_N_4_ and 1-RPh-g-C_3_N_4_. This might be due to the addition of phosphorus in the g-C_3_N_4_ skeleton, which can lead to an enhanced surface area and capacitance. In all the CV voltammograms, a broad anodic/cathodic peak was observed which corresponds to the pyridinic nitrogen redox reaction with the electrolyte, and is in accordance with similar previous reports^10^. For a more detailed study, the specific capacitance of the B-g-C_3_N_4_, 1-RPh-g-C_3_N_4_, and 2-RPh-g-C_3_N_4_ heterostructures were calculated by considering the integrated area of the representative CV voltammograms using the equation reported elsewhere^47^,^48^,^49^. The estimated specific capacitance of B-g-C_3_N_4_, 1-RPh-g-C_3_N_4_, and 2-RPh-g-C_3_N_4_ heterostructure were 63.37, 178.53, and 241.83 Fg^−1^, respectively, at a scan rate of 100 mVs^−1^.

Furthermore, the CV measurements of B-g-C_3_N_4_, 1-RPh-g-C_3_N_4_, and 2-RPh-g-C_3_N_4_ heterostructure were also examined at different scan rates, which are shown in Fig. 5b–d. As evident from the capacitance calculation and CV profile of all the samples, the 2-RPh-g-C_3_N_4_ heterostructure showed the highest capacitance with a larger capacitive area, which may be due to the high surface area and modified electronic skeleton of the carbon nitride by the addition of phosphorus.

**Figure 6:**
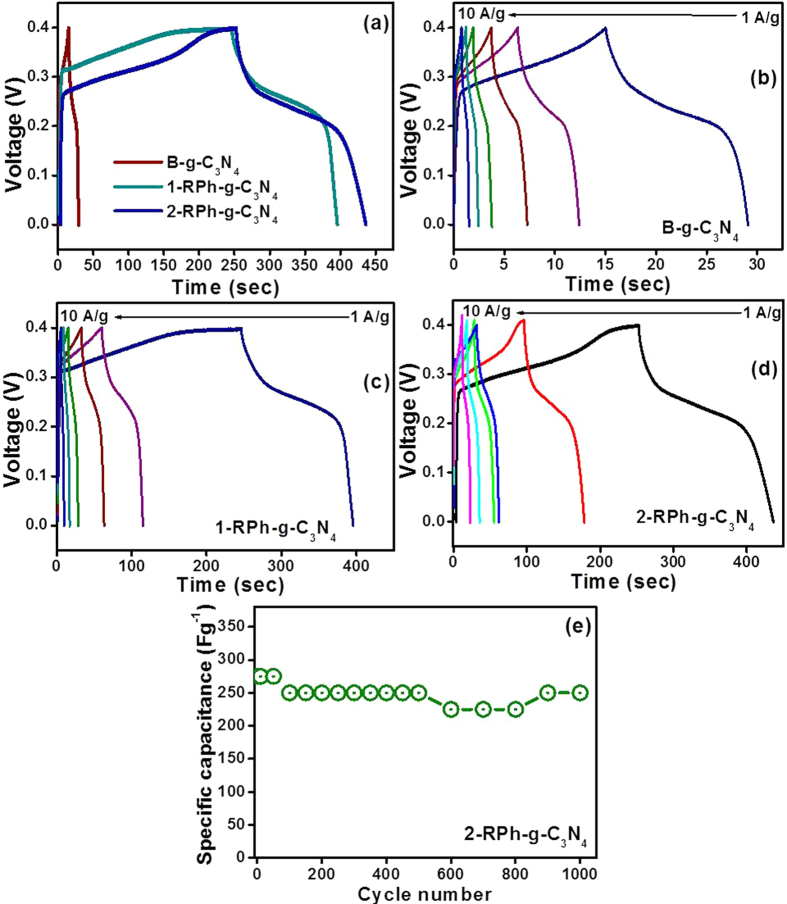
(**a**) Comparative galvanostatic CD profile of B-g-C_3_N_4_, 1-RPh-g-C_3_N_4_, and 2-RPh-g-C_3_N_4_ heterostructure electrode at a current load of 10 Ag^−1^, (**b**) Galvanostatic CD curves of B-g-C_3_N_4_ at a current load of 1–10 Ag^−1^, (**c**) Galvanostatic CD curves of 1-RPh-g-C_3_N_4_ at a current load of 1–10 Ag^−1^, (**d**) Galvanostatic CD curves of 2-RPh-g-C_3_N_4_ heterostructure at a current load of 1–10 Ag^−1^, and (**e**) Cyclic stability of the 2-RPh-g-C_3_N_4_ heterostructure at a current load of 10 Ag^−1^.

To better understand the capacitive behavior of B-g-C_3_N_4_, 1-RPh-g-C_3_N_4_, and 2-RPh-g-C_3_N_4_ heterostructure, the most valuable galvanostatic charge-discharge (GCD) approach was conducted and the specific capacitance of all the materials was calculated using the previously reported equation^13^,^14^,^15^,^16^,^50^. Figure 6a presents the GCD profile of the B-g-C_3_N_4_, 1-RPh-g-C_3_N_4_, and 2-RPh-g-C_3_N_4_ at a fixed current load, whereas Fig. 6b–d represents the comparative GCD curve of the B-g-C_3_N_4_, 1-RPh-g-C_3_N_4_, and 2-RPh-g-C_3_N_4_ heterostructure, at different current loads. In the Fig. 6a, the comparative GCD profile shows that the 2-RPh-g-C_3_N_4_ exhibits a short charging time and a longer discharging time performance as well as higher specific capacitance than B-g-C_3_N_4_, and 1-RPh-g-C_3_N_4_ heterostructure at 10 Ag^−1^, which further highlights the combined contribution of RPh and g-C_3_N_4_.

The estimated specific capacitance of the B-g-C_3_N_4_ at 1, 2, 3, 5, 7, and 10 Ag^−1^ current loads were 35.22, 30.40, 27, 23.25, 21.7, and 16 F g^−1^, respectively. Similarly, the specific capacitance obtained for 1-RPh-g-C_3_N_4_ at a current of 1, 2, 3, 5, 7, and 10 Ag^−1^ was 372.5, 270, 225.82, 167.12, 137.2, and 97 F g^−1^, respectively, whereas the specific capacitance of the 2-RPh-g-C_3_N_4_ heterostructure at a current of 1, 2, 3, 5, 7, and 10 Ag^−1^ were 465, 405, 225, 350, 297.5, and 275 F g^−1^, respectively. Interestingly, the above results measured at different current loads shows that the specific capacitance of the 2-RPh-g-C_3_N_4_ heterostructure is much higher than bare materials, which might be due to the synergistic effects of the two constituents in the resulting heterostructure.

Furthermore, the possibility of the long term use of the as-prepared capacitor electrode was examined by a repeated charge-discharge cycling test because the long term stability of the electrode material is essential for its practical applications. Figure 6e shows the results of the GCD cycling test of the 2-RPh-g-C_3_N_4_ heterostructure at a current load of 10 Ag^−1^ and a potential range of 0–0.4 V for 1000 cycles. Even after 1000 cycles and harsh continuous loads, the 2-RPh-g-C_3_N_4_ heterostructure electrode retained 90% of capacitance after 1000 continuous cycles.

The dramatically enhanced electrochemical supercapacitive performance of the 2-RP-g-C_3_N_4_ heterostructure can be attributed to various factors, such as the presence of nitrogen, which provides a large number of active sites, improved donor/acceptor characteristics of the electrons and additional pseudocapacitance. In addition, the presence of RPh in the heterostructure skeleton plays decisive roles in enhancing the capacitive performance of the as-prepared heterostructure by combining with the C/N framework of the g-C_3_N_4_, which are helpful in enhancing the conductivity by accelerating the electron transfer process. Thus, due to the synergistic effect of the RPh and g-C_3_N_4_, the as-prepared heterostructure exhibited remarkably enhanced electrochemical supercapacitive performance.

## Conclusions

RPh-g-C_3_N_4_ heterostructures were synthesized via a facile and inexpensive ball milling approach using RPh and melamine, as the starting materials. The optimized RPh-g-C_3_N_4_ heterostructure displayed significantly enhanced visible light-driven photocatalytic activity and supercapacitive behavior compared to the bare materials. The interaction of the constituents of the heterojunction at the interface i.e. the interaction between RPh and g-C_3_N_4_ is the prime reason for the improved separation of the photogenerated charge carriers which thereby reduces the possibility of charge carrier recombination during visible light photoirradiation, and is proved further by the electrochemical impedance spectroscopy. The as-prepared RPh-g-C_3_N_4_ heterostructure under the optimized reaction conditions showed better supercapacitance performance of 465 F g^−1^ compared to the other bare materials. The excellent electrochemical supercapacitive behavior of the RPh-g-C_3_N_4_ heterostructure is due to the combined synergistic effects of RPh (which induces the capacitance) and g-C_3_N_4_ (which provide a high surface area and nitrogen skeleton). In other words, the excellent visible light photocatalytic behavior, photoelectrochemical performance as a photoelectrode and electrochemical capacitive performance as a supercapacitor electrode of RPh-g-C_3_N_4_ heterostructure are due mainly to the unique properties of the constituent materials, such as the high surface area, narrow band gap energy, capacitive behavior, and high nitrogen content.

### Materials and analysis methods

The model organic pollutant, Rhodamine B (RhB), was acquired from Sigma–Aldrich and red phosphorus (RPh) was purchased from Yakuri Pure Chemicals, Kyoto Japan. Ethyl cellulose and α-terpineol were supplied by KANTO Chemical Co., Japan. Fluorine-doped transparent conducting oxide glass (FTO; F-doped SnO_2_ glass; 7 Ω/sq) was acquired from Pilkington, USA, whereas sodium sulfate (Na_2_SO_4_), methyl orange (MO) and 4-nitrophenol (4-NP) were obtained from Duksan Pure Chemicals Co. Ltd. South Korea. Nickel foam with >99.99% purity was purchased from MTI Corporation, USA (thickness 1.6 mm, surface density 346 g m^−2^ and porosity ≥95%). All other chemicals used in this study were of analytical grade and used as received. The deionized water obtained from a PURE ROUP 30 water purification system was used to prepare the required solutions.

## Methods

The purity and structural characterization was accomplished by X-ray diffraction (XRD, PANalytical, X’pert PRO-MPD, Netherland) using Cu Kα radiation (λ = 0.15405 nm). Ultraviolet-visible-near infrared (UV-VIS-NIR, Cary 5000, VARIAN, USA) spectrophotometry equipped with a diffuse reflectance integrating sphere was used to examine the light absorption properties of the as-prepared photocatalysts. The charge recombination of photoinduced electron and holes was recorded by photoluminescence (PL, Kimon, 1 K, Japan) spectroscopy over the scanning range, 200–800 nm, with an excitation wavelength of 325 nm. PL was conducted at the Korea Basic Science Institute, Gwangju Center, South Korea. X-ray photoelectron spectroscopy (XPS, ESCALAB 250 XPS System, Thermo Fisher Scientific U.K.) was performed using monochromatized Al Kα x-rays (hν = 1486.6 eV) to understand the chemical interactions among the constituents and the surface composition of the samples. The size and morphology of the as-synthesized photocatalysts were observed by field emission transmission electron microscopy (FE-TEM, Tecnai G2 F20, FEI, USA) operated at an accelerating voltage of 200 kV. The surface area of the samples were measured using the Brunauer–Emmett–Teller (BET) method (ASAP 2020, Physisorption Analyzer Micromeritics Inc. USA) using the N_2_ adsorption-desorption isotherms. A 400 W visible light lamp with an intensity of 31 mW/cm^2^ and λ > 500 nm obtained from 3M USA was used as the light source for the photocatalytic and photoelectrochemical studies. The photoelectrochemical and electrochemical supercapacitance experiments were carried out using a potentiostat (VersaSTAT 3, Princeton Research, USA) equipped three electrode assembly cell. A platinum sheet and Ag/AgCl (3.0 M KCl) were used as the counter and reference electrodes, respectively.

### Mechanical ball milling of commercial RPh and g-C_3_N_4_

#### Preparation of P-g-C_3_N_4_

The direct heating method reported in literature procedures was used to synthesize g-C_3_N_4_^22^,^24^,^25^. Briefly, 5 mg of commercial melamine was placed in a quartz container, heated from room temperature to 500 °C and stabilized for 2 h under a constant N_2_ flow. After cooling the furnace tube to room temperature, light yellow g-C_3_N_4_ was collected, which was grinded into a fine powder and stored for further characterization and other analysis.

The as-prepared g-C_3_N_4_ (P-g-C_3_N_4_) was used to synthesize the RPh-g-C_3_N_4_ photocatalyst heterostructures, in which commercially available RPh at different weight percentages was mixed with g-C_3_N_4_ using a simple ball milling method. The g-C_3_N_4_ with 10 (1-RPh-g-C_3_N_4_) and 30 (2-RPh-g-C_3_N_4_) wt.% RPh were prepared by milling the above mixture for 12 h at 400 rpm. For a detailed comparative study, a similar composition of RPh and g-C_3_N_4_ was also milled for 24 h at 400 rpm and were referred to as 3-RPh-g-C_3_N_4_ and 4-RPh-g-C_3_N_4_. The pure g-C_3_N_4_ (P-g-C_3_N_4_) was also milled under similar conditions for the comparative study and was abbreviated as B-g-C_3_N_4_.

#### Photodegradation test

The visible light photodegradation test was carried out by choosing commercially available model organic colored pollutants, such as RhB and MO, as well as non-colored organic compound i.e., 4-NP. In a typical degradation procedure, 3 mg of the prepared catalyst was dispersed in 20 mL of an aqueous solution of RhB (5 mg/L), 20 mL MO (5 mg/L), and 20 mL 4-NP (5 mg/L) and subsequently stirred magnetically for 30 min in the dark to achieve adsorption-desorption equilibrium. The above photocatalyst suspensions were then irradiated with visible light. After a desired irradiation time, 2 mL sample was taken and the catalyst was separated by centrifugation to obtain a clear liquid and the absorption spectra of the clear liquid were further recorded by using a UV–vis spectrophotometer. The recorded absorption spectra were used further to calculate the degradation rate using the method reported elsewhere^4^,^5^.

### Photoelectrode preparation and its visible light driven photoelectrochemical studies

A similar procedure to that reported previously was used to prepare the working electrodes^4^. Typically, an appropriate amount of the photocatalyst (100 mg) was suspended in ethyl cellulose and α-terpineol followed by proper mixing by sonication and stirring to obtain a fine paste. The resulting paste was then coated using a blade on a FTO glass electrode on an effective area of 1 cm^2^ and dried further in a commercially available drying lamp. EIS measurements of the representative photocatalysts electrode were taken in an aqueous electrolyte (0.2 M Na_2_SO_4_) in the dark and under visible light photoirradiation. EIS analysis was carried out at a potential of 0.0 V in the dark and under visible photoirradiation at frequencies of 1 to 10^4^ Hz.

### Electrode fabrication and its electrochemical supercapacitance measurements

A previously reported methodology was followed for the preparation of the electrode on commercially available nickel foam^51^. In a typical process, in a mixture of ethanol and Nafion solutions, 1 mg of the representative heterostructures and bare materials samples were dispersed by sonication to obtain a homogeneous slurry. The resulting slurry was then coated on the nickel foam with an effective area of 1 cm^2^, further dried under a lamp and thereafter used as the working electrode. The CV and galvanostatic CD measurements were used to evaluate the electrochemical capacitance of the RPh-g-C_3_N_4_ heterostructures as supercapacitor electrode materials.

## Additional Information

**How to cite this article**: Ansari, S. A. *et al*. Facile and Scale Up Synthesis of Red Phosphorus-Graphitic Carbon Nitride Heterostructures for Energy and Environment Applications. *Sci. Rep.* **6**, 27713; doi: 10.1038/srep27713 (2016).

## Supplementary Material

Supplementary Information
