# Supplementary material for: Facile and Scale Up Synthesis of Red Phosphorus-Graphitic Carbon Nitride Heterostructures for Energy and Environment Applications
Source: Sci Rep. 2016 Jun 13;6:27713. doi: 10.1038/srep27713 (PMC4904207; doi:10.1038/srep27713)
Supplement: Supplementary Information [file srep27713-s1.pdf]

Supporting Information

Facile and Scale Up Synthesis of Red Phosphorus-Graphitic Carbon Nitride Heterostructures for Energy and Environment Applications

Sajid Ali Ansari[1*], Mohammad Omaish Ansari[2] and Moo Hwan Cho[1*]

[1]School of Chemical Engineering, Yeungnam University, Gyeongsan-si, Gyeongbuk 712-749, South Korea, Phone: +82-53-810-2517; Fax: +82-53- 810-4631.

[*]Corresponding authors: mhcho@ynu.ac.kr, sajidansari@ynu.ac.kr

[2]Center of Nanotechnology, King Abdulaziz University, Jeddah 21589, Saudi Arabia.

UV-vis Diffuse reflectance spectra of the P-g-C_3N_4, B-g-C_3N_4, 1-RPh-g-C_3N_4, and 2-RPh-g-C_3N_4 heterostructure

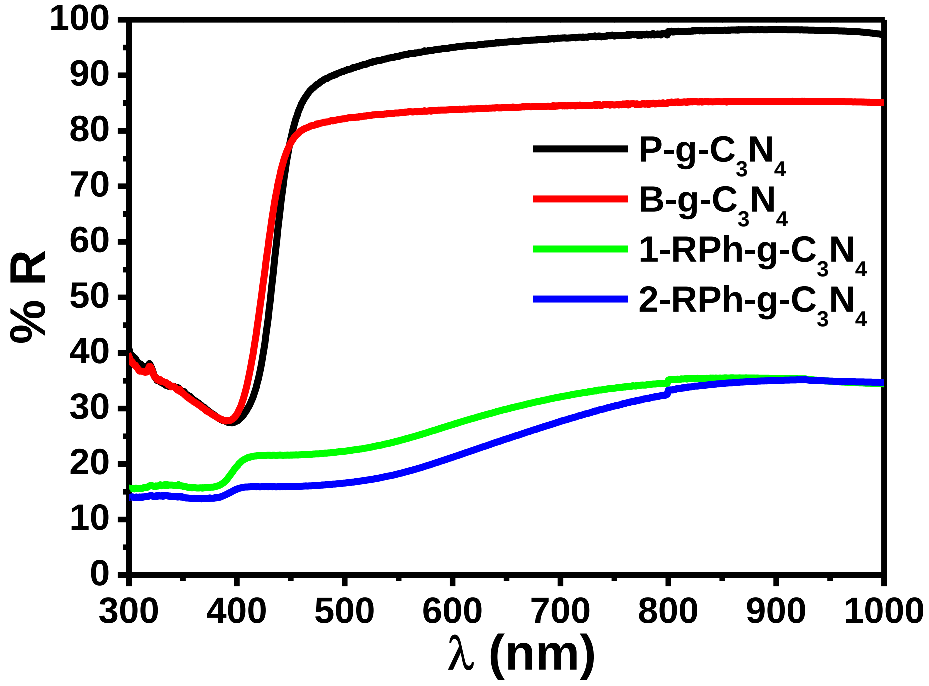

Figure S1. UV-vis Diffuse absorption spectra of the P-g-C_3N_4, B-g-C_3N_4, 1-RPh-g-C_3N_4, and 2-RPh-g-C_3N_4 heterostructure.

TEM Image of the B-g-C_3N_4

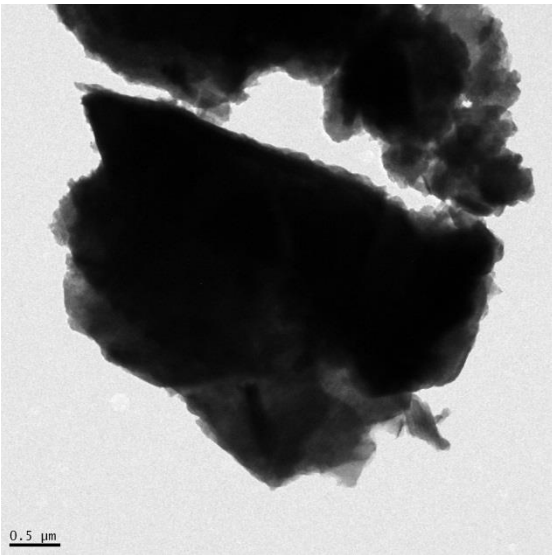

Figure S2. TEM image of the B-g-C_3N_4.

TEM Image of the B-g-C_3N_4

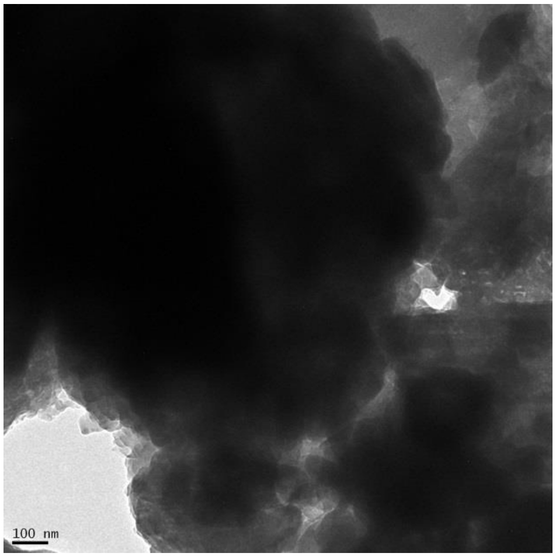

Figure S3. TEM image of the B-g-C_3N_4.

XPS survey spectra

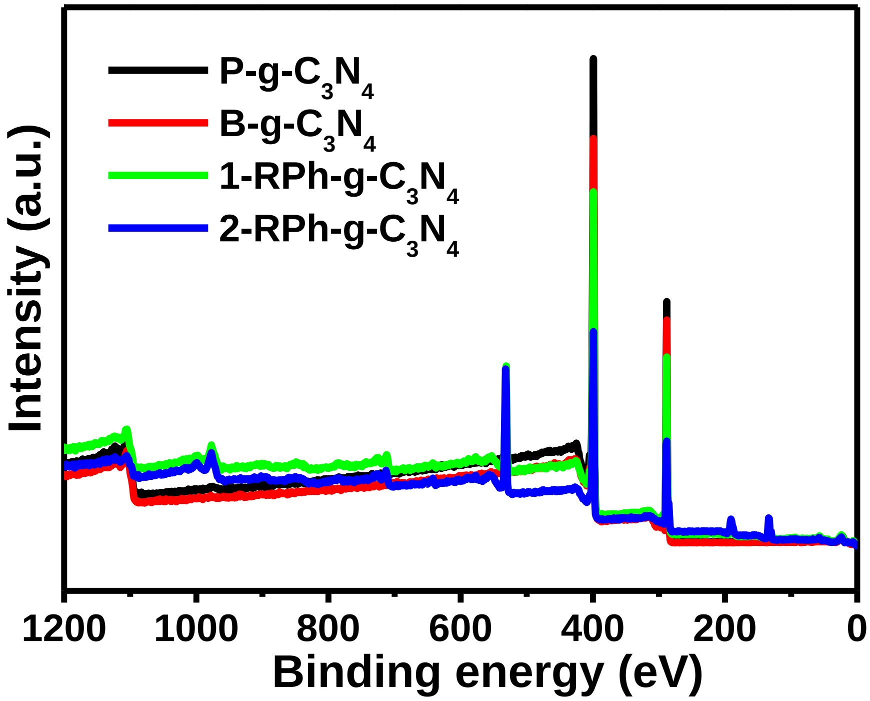

Figure S4. XPS survey spectra of the P-g-C_3N_4, B-g-C_3N_4, 1-RPh-g-C_3N_4, and 2-RPh-g-C_3N_4 heterostructure.

High resolution C 1s core level spectra of P-g-C_3N_3, B-g-C_3N_3, 1-RPh-g-C_3N_3, and 2-RPh-g-C_3N_3 heterostructure

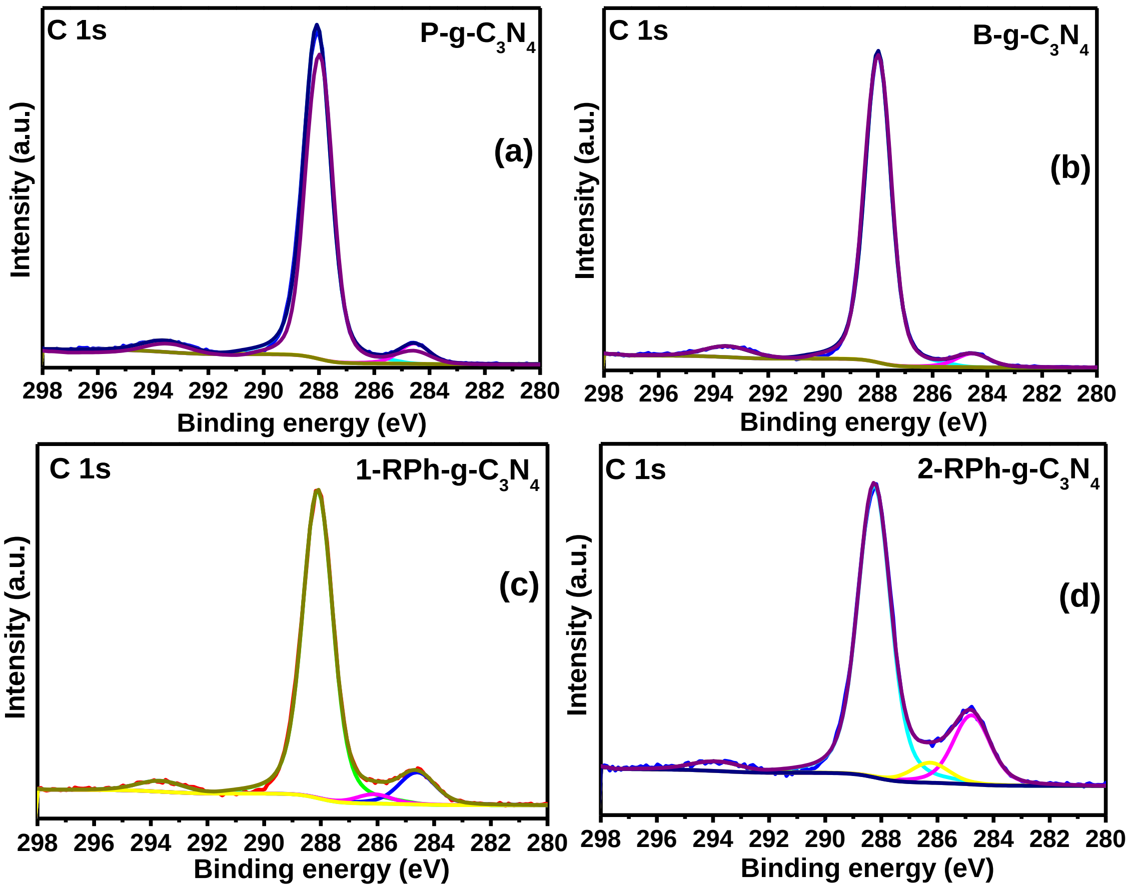

Figure S5**.** High resolution C 1s core level spectra of (**a**) P-g-C_3N_4, (**b**) B-g-C_3N_4, (**c**) 1-RPh-g-C_3N_4, (**d**) 2-RPh-g-C_3N_4 heterostructure.

Comparative degradation kinetic plot of MO and RhB under photoirradiation in the presence of photocatalysts

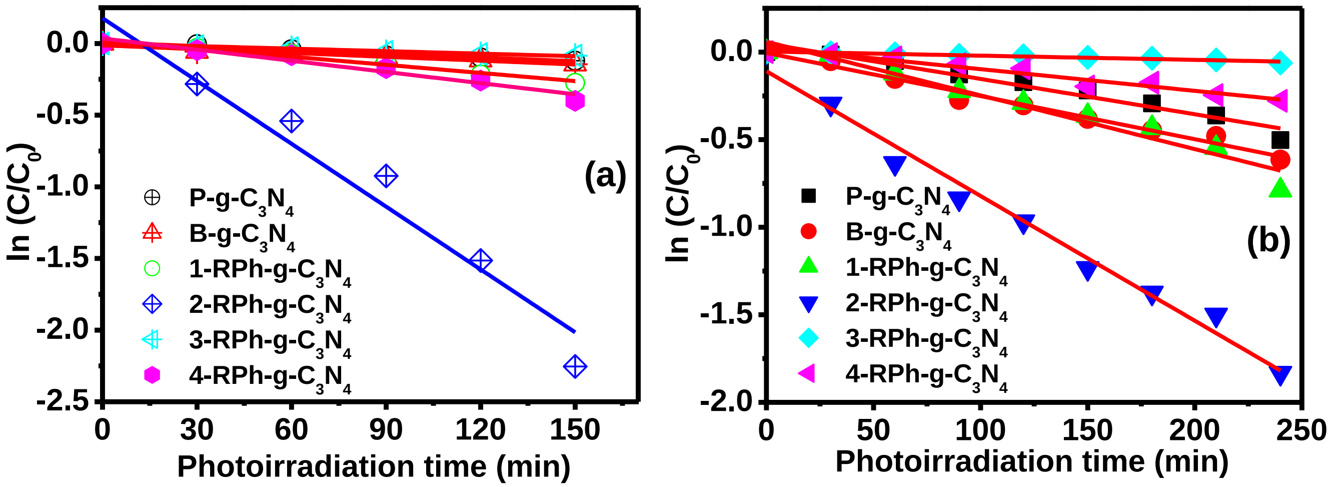

Figure S6. The C/C_0 versus photoirradiation time plots for (**a**) MO and (**b**) RhB degradation over the P-g-C_3N_4, B-g-C_3N_4, 1-RPh-g-C_3N_4, 2-RPh-g-C_3N_4, 3-RPh-g-C_3N_4, 4-RPh-g-C_3N_4 heterostructures.

Photodegradation kinetic plot of 4-NP as the function photoirradiation time

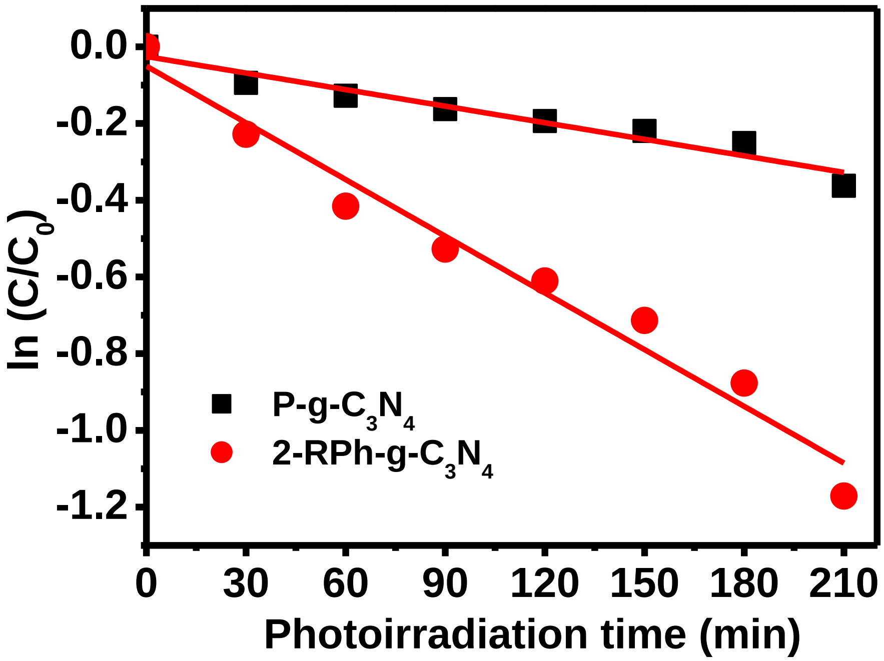

Figure S7. Plot of ln C/C_0 vs the photoirradiation time for the photodegradation of 4-NP by P-g-C_3N_4 and 2-RPh-g-C_3N_4 heterostructure.
